# Identification of Additional Cases of Severe Neonatal GABA‐Transaminase Deficiency

**DOI:** 10.1002/jmd2.70069

**Published:** 2026-01-20

**Authors:** Deima Alammary, Tisiana Low, Ganesh Srinivasan, Marie‐Claude Dery, Mahmoud Almutadares, Samantha Marin, Mubeen F. Rafay, Katya Rozovsky, Patrick Frosk

**Affiliations:** ^1^ Sections of Neonatology, Department of Pediatrics and Child Health, Rady Faculty of Health Sciences University of Manitoba Winnipeg Manitoba Canada; ^2^ Genetics and Metabolism, Department of Pediatrics and Child Health, Rady Faculty of Health Sciences University of Manitoba Winnipeg Manitoba Canada; ^3^ Clinical Biochemistry Diagnostic Services, Shared Health Winnipeg Manitoba Canada; ^4^ Department of Genetic Medicine, Faculty of Medicine King Abdulaziz University Jeddah Saudi Arabia; ^5^ Neurology, Department of Pediatrics and Child Health, Rady Faculty of Health Sciences University of Manitoba Winnipeg Manitoba Canada; ^6^ Diagnostic Imaging, Department of Pediatrics and Child Health, Rady Faculty of Health Sciences University of Manitoba Winnipeg Manitoba Canada

**Keywords:** burst suppression, founder mutation, GABA transaminase deficiency, neonatal encephalopathy, urine GABA quantification

## Abstract

GABA‐transaminase (GABA‐T) deficiency is a rare disorder of GABA metabolism characterized by neonatal encephalopathy, epilepsy, hypotonia and intellectual disability. It is caused by biallelic pathogenic variants in the *ABAT* gene. We report a case of a newborn female born to a G10P5 mother, with abnormal fetal movements and polyhydramnios in utero. At birth, she presented with hypotonia, hypersomnolence, decreased level of consciousness, central hypoventilation, non‐epileptic myoclonus, seizures, and neurogenic diabetes insipidus. Brain MRI on day two of life showed partial cerebellar vermis agenesis and cerebellar hemispheric dysplasia. Her EEG demonstrated burst suppression. Family history was significant for two siblings with a similar neonatal course. On rapid whole exome sequencing she was found to be homozygous for a nonsense variant in the *ABAT* gene designated c.1278C>A, p.Tyr426*. Both of her affected siblings were also found to be homozygous for the same variant, and carrier status was confirmed in both parents. A trial of flumazenil infusion showed subtle EEG improvement. Our report of three siblings with severe GABA‐T deficiency provides evidence for founder effect in the Canadian Indigenous population and discusses the utility of urine GABA quantification as a reasonable screening test.

## Synopsis

A case report showing that GABA‐T deficiency can present quite severely in neonates with early lethality and there may be utility in using urine GABA quantification as a readily accessible screening test.

## 1 Introduction

GABA‐transaminase (GABA‐T) deficiency (OMIM#613163) is a rare disorder of GABA metabolism characterized by encephalopathy, hypotonia, developmental delay and epilepsy, presenting in the neonatal period or early infancy [1] Gamma‐aminobutyric acid (GABA) is a primary inhibitory neurotransmitter of the central nervous system. GABA is degraded to succinic semialdehyde by the enzyme GABA transaminase via the GABA shunt pathway of the tricarboxylic acid cycle [2]. GABA‐T deficiency is caused by homozygous or compound heterozygous mutations in the *ABAT* gene (OMIM#137150) [3]. Due to the rarity of cases of GABA‐T deficiency ascertained worldwide, the diagnosis is made with a combination of clinical, molecular and biochemical testing [4]. In current practice, demonstration of elevated levels of GABA in plasma, urine and CSF, as well as a GABA peak on magnetic resonance spectroscopy (MRS), are supportive of this disorder [1, 5].

## 2 Case Report

**FIGURE 1:**
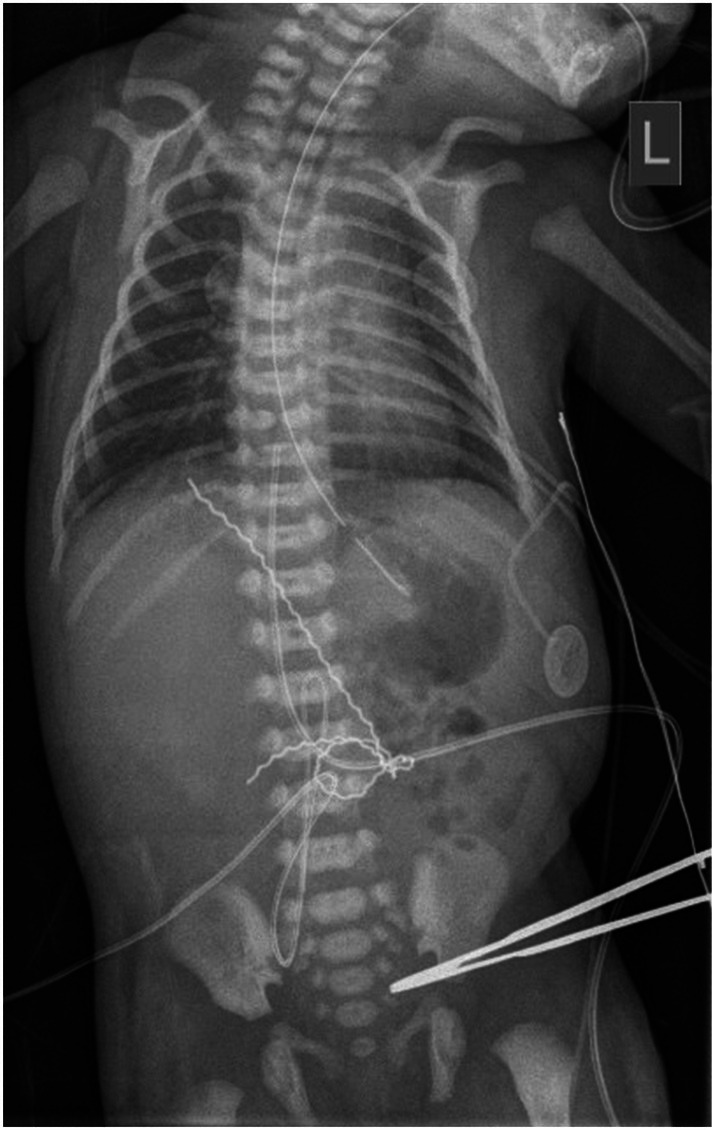
Chest X‐ray on day 1 of life revealing perihilar and lower lobe atelectasis bilaterally, and an incidental finding of T8 butterfly vertebra.

We present a newborn female born to a G10P5SA4 mother at 36 weeks and 6 days of gestation. The pregnancy was complicated by polyhydramnios, lack of fetal movements, and persistent abnormal hand positioning (hands clenched with extended 2nd and 5th digits) on antenatal ultrasound. She was delivered by caesarean section due to non‐reassuring fetal heart tracing. At birth, her growth parameters were appropriate for 36 weeks gestational age. The birth weight, length, and head circumference were 2450 g (10th–50th percentile), 47 cm (10th–50th percentile), and 33 cm (50th percentile), respectively. Her Apgar scores were 4, 6, and 7 at 1, 5, and 10 min of life, respectively. She required mechanical ventilation within the first hour of life due to insufficient respiratory efforts. The neurological examination demonstrated a decreased level of consciousness with poor reactivity to external stimulation, pupils unreactive to light, absent gag response, diffuse hypotonia, minimal spontaneous movements, hyporeflexia, and absent neonatal primitive reflexes. Apart from micro‐retrognathia and low set ears, the infant had no dysmorphic features. Her course in the neonatal intensive care unit was complicated by the development of neurogenic diabetes insipidus (DI), treated with hydrochlorothiazide, free water replacement, and a low‐sodium formula. She developed hypotension requiring inotropic support. A chest X‐ray performed on day 1 revealed mild bilateral perihilar and lower lobe atelectasis, a normal cardio‐thymic silhouette, and a T8 butterfly vertebra (Figure 1). Frequent multifocal myoclonic jerks were noted at birth, which initially responded to phenobarbital but subsequently returned and were highly stimulus‐sensitive.

**FIGURE 2:**
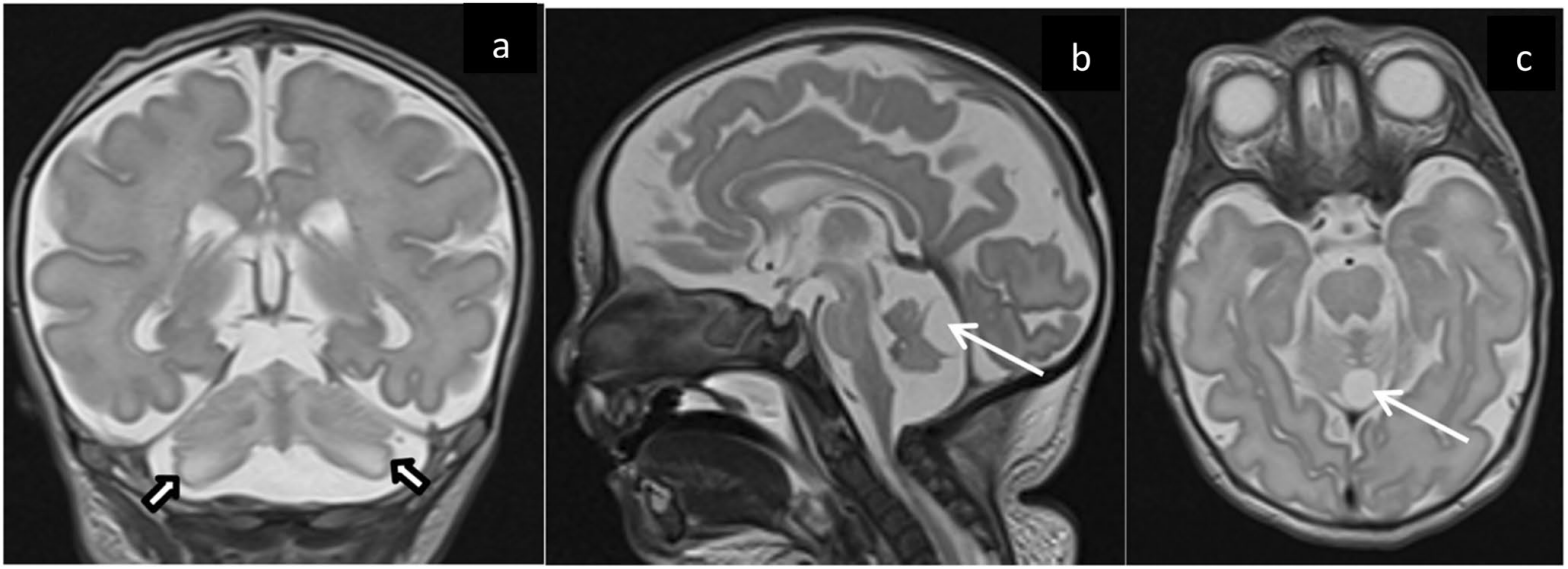
Brain MRI obtained at day 2 of life. T2 weighted coronal (a), sagittal (b), and axial (c) images of the brain: The cerebellar hemispheres and vermis appear dysplastic (hollow arrows). There is a cleft involving the posterior aspect of the vermis, with adjacent thin‐walled fluid‐filled cyst (arrow). The findings are in keeping with partial vermian agenesis. No evidence of diffusion restriction or bleeding (not shown).

**FIGURE 3:**
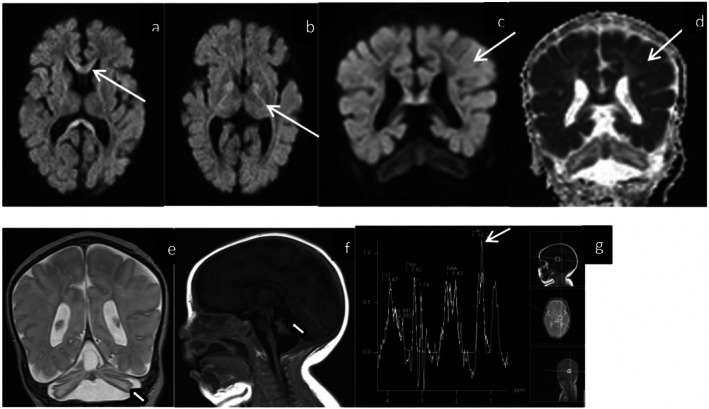
Follow‐up brain MRI for index patient obtained at day 48 of life. Axial (a, b) and coronal (c) DWI weighted images, coronal (d) ADC‐map: There is a new restricted diffusion within the cortex and subcortical white matter of both cerebral hemispheres, corpus callosum, internal capsule, and corticospinal tracts bilaterally and symmetrically (arrows). Coronal T2 (e) and sagittal T1 (f) weighted images: Again visualized is a partial vermian agenesis with a cyst along its dorsal aspect (hollow arrows). MRS spectroscopy (G) demonstrates lactate peak (arrow) within the left‐sided basal ganglia. Abbreviation: MRI, magnetic resonance imaging.

Her electroencephalography (EEG) initially revealed a burst‐suppression pattern, suggestive of severe brain dysfunction. The myoclonic jerks had no electrographic correlate and were consistent with non‐epileptic myoclonus. There were two electroclinical seizures captured, one with right chin and left eye twitching lasting up to 2 min and the other with subtle right chin twitching lasting a few seconds. Her seizures were treated with phenobarbital and levetiracetam, but the myoclonus was poorly responsive to treatment. Later in her course, her EEG tracing was consistent with electrocerebral silence. Magnetic resonance imaging (MRI) of the brain on day 2 of life showed bilateral cerebellar hemispheric and vermis dysplasia, and a cleft involving the posterior aspect of the vermis with adjacent thin‐walled fluid‐filled cyst (Figure 2). A follow‐up MRI brain on day 48 revealed new symmetrical and bilateral restricted diffusion within the cortex and subcortical white matter of cerebral hemispheres, and within the corpus callosum, internal capsule and corticospinal tracts. MRS demonstrated a lactate peak noted within the left‐sided basal ganglia on (Figure 3).

**FIGURE 4:**
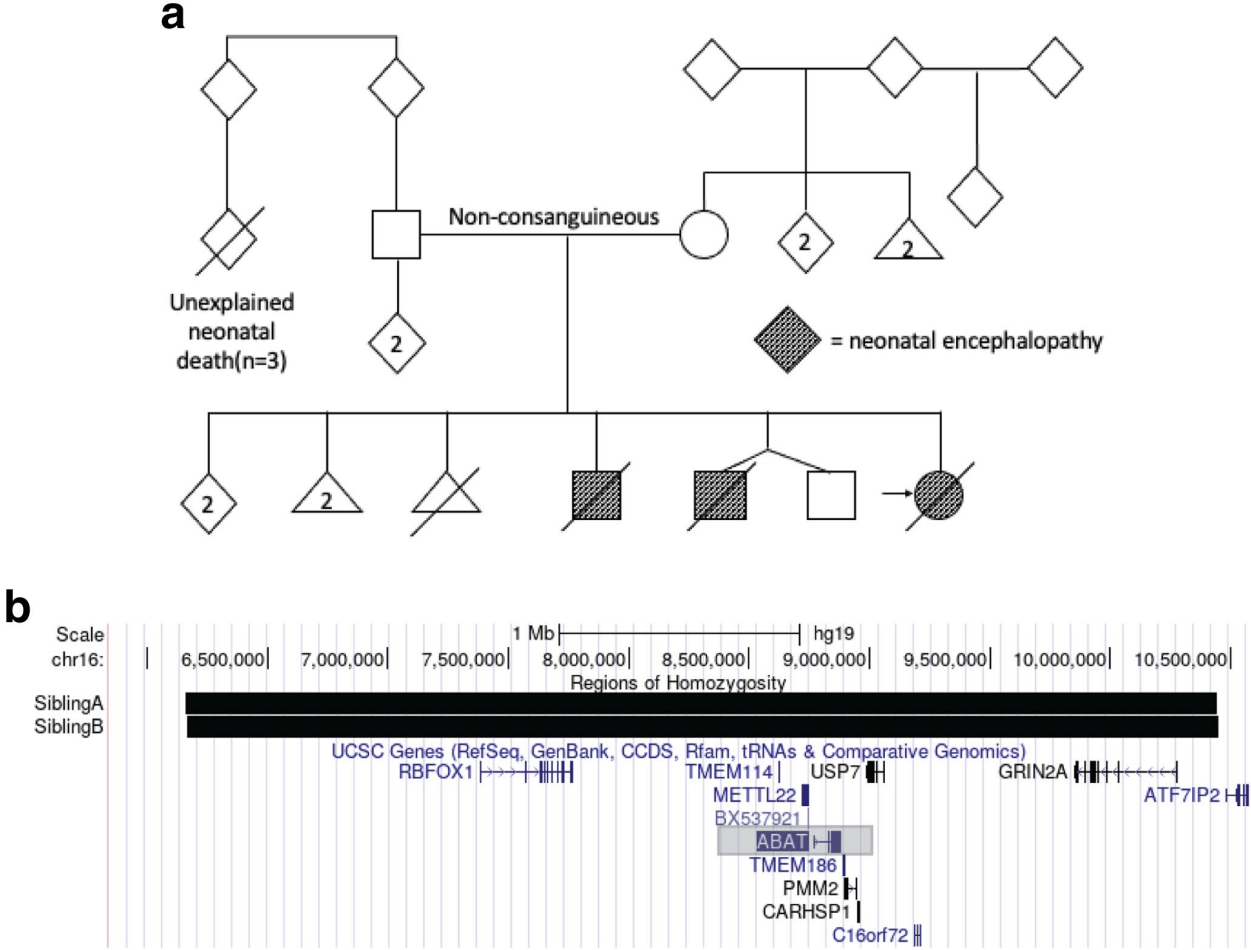
(a) Pedigree for the reported family using standard nomenclature. (b) Genome browser showing the segment of chromosome 16 containing the ABAT gene (highlighted in grey box). Depicted are the surrounding regions of homozygosity identified on clinical SNP microarray (Invitae, California, US) in Siblings (a) and (b) (genome build hg19).

Parents were of Canadian Indigenous descent, with no history of consanguinity (Figure 4). Family history included two previous male infant deaths within the family at 9 and 21 days in the neonatal period. The first neonatal death was a late preterm, born at 35 weeks of gestational age (Sibling A). He was noted to have dysmorphic features involving a small anterior fontanelle, hypertelorism, high nasal root, downslanting palpebral fissures and simple ear helices, retrognathia, single palmar crease on the right, overriding digits, a micropenis, and an abnormal neurological examination with hypertonia and frequent myoclonus. Brain MRI showed hypoplastic cerebellar hemispheres and vermis, with decreased myelination of the brainstem. EEG demonstrated burst suppression. The family opted for comfort care and the infant died subsequently at 9 days of age. Whole exome sequencing was not performed for this infant. The second neonatal death was a dichorionic diamniotic preterm twin born at 32 weeks of gestational age (Sibling B). The delivery was complicated by severe metabolic acidosis, hypotension, and respiratory failure. He had focal seizures which were treated with phenobarbital and levetiracetam. His brain MRI showed intraparenchymal and intraventricular hemorrhage, immature gyration and myelination with hypoplastic cerebellar hemispheres. The clinical presentation indicated severe brain injury, and the family decided to withdraw respiratory support at 21 days of life.

A single unifying autosomal recessive diagnosis for all three siblings seemed likely. Nondiagnostic microarray analysis had previously been completed on the two deceased siblings so this was not pursued in the index case. A large gene panel was arranged, targeting genes involved in epileptic encephalopathy, which was unrevealing. Subsequently, rapid exome sequencing was performed by Prevention Genetics, and a homozygous nonsense variant in the *ABAT* gene was identified (c.1278C>A; p.Tyr426*). The nonsense variant is predicted to result in a complete loss of function via nonsense mediated decay based on its position in the gene. Targeted testing for the *ABAT* c.1278C>A was performed in the family members. The deceased siblings were found to be homozygous for the same variant, and the parents were confirmed to be heterozygous carriers. At the time of genetic testing, we noted that there was no observation for this variant in gnomAD and that this variant was not present in ClinVar, leading to the variant being classified as a variant of uncertain significance (VUS). This variant was recently reclassified as “Likely Pathogenic” in ClinVar on October 8, 2024.

In light of the exome findings, multiple efforts were made to obtain independent biochemical evidence for elevated GABA levels. Unfortunately, the detection of brain GABA peak via MRS was not technically possible. Plasma and urine amino acids were arranged early in her course and were found to be elevated in a non‐specific pattern. Plasma AA showed GABA at 12 and 15 μmol/L (reference range < 4 nmol/mL) in two samples 3 weeks apart.

**FIGURE 5:**
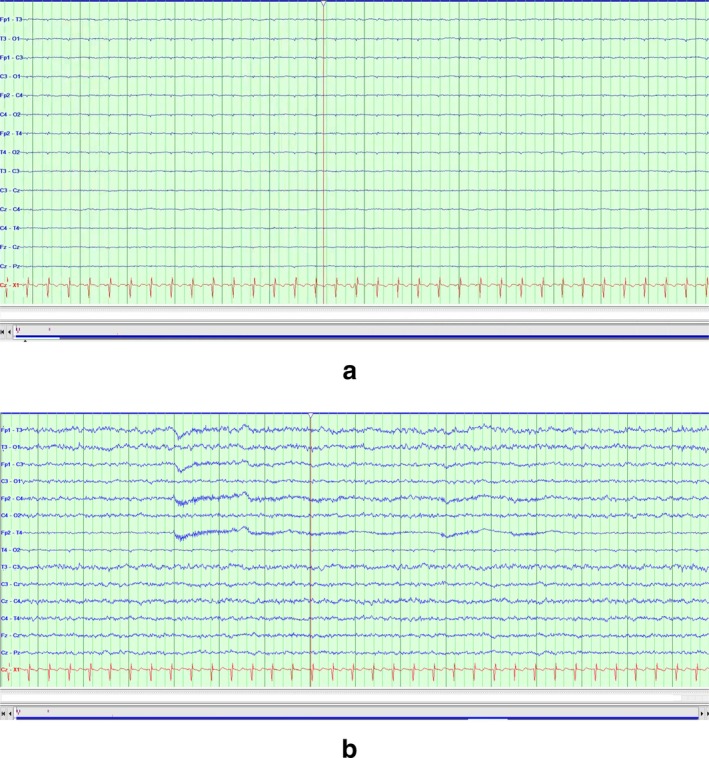
(a) EEG prior to treatment showing complete attenuation of the background activity (LFF 1 Hz, HFF 70 Hz, notch 60 Hz, sensitivity 7 μV/mm, timebase 30 mm/s). (b) EEG After treatment showing low amplitude mixed frequencies (LFF 1 Hz, HFF 70 Hz, notch 60 Hz, sensitivity 7 μV/mm, timebase 30 mm/s).

Urine AA detected GABA at 562 μmol/mmol creatinine. However, GABA is not generally reported by our laboratory since it is rarely applicable to most inborn errors of metabolism. Subsequent analysis of these profiles after completion of genetic testing revealed serum GABA levels were increased to 74 nmol/mL (reference range < 4 nmol/mL) and, in the urine, GABA excretion was significantly elevated at 5403 nmol/mg creatinine (reference range < 25 nmol/mg creatinine). 4‐Hydroxybutyric acid, the immediate precursor of GABA, was not detected by organic acid analysis. Lumbar puncture for CSF measures of GABA was attempted thrice without success. Her family consented for a flumazenil trial with concomitant continuous EEG monitoring. The EEG prior to flumazenil administration revealed electrocerebral silence (Figure 5a). She was initiated on a flumazenil bolus of 0.01 mg/kg/dose, followed by an infusion with a rate of 0.05 mg/kg/h overnight. EEG showed low voltage slowing which was a change from electrocerebral silence and subtle clinical improvement indicated by appearance of mild gag reflex within 24 h of flumazenil infusion (Figure 5b). Due to the minimal improvement, her family elected for non‐invasive palliative/comfort care at that point and withdrawal of mechanical ventilation was carried out at 51 days of life with resultant infant death.

## 3 Discussion

The first case of GABA‐transaminase deficiency was reported in 1984 with a clinical presentation of pronounced hypotonia, generalized hyperreflexia, convulsions and psychomotor retardation [3]. Increased levels of GABA were found in the CSF and urine, and a liver biopsy revealed deficiency of α‐aminobutyric acid transaminase activity [3]. Since then, at least 17 cases have been reported worldwide with variable phenotype, but the main features include neurodevelopmental impairment, seizures and hypersomnolence [1]. GABA‐transaminase deficiency is inherited in an autosomal recessive fashion, via either homozygous or compound heterozygous variations of the *ABAT* gene (OMIM 137150). The most striking finding was the cerebellar vermis hypoplasia noted in the index case and also documented in her siblings. In our case report, all three siblings had severe clinical presentations with marked encephalopathy, characteristic mutually shared cerebellar abnormalities on neuroimaging and early lethality. There is also a history of two spontaneous abortions in the family. Although postmortem examination was not performed, this observation raises the possibility of early fetal demise in this condition. Previous *ABAT* variants in the literature have been associated with loss of function missense or complete gene deletion in heterozygous fashion [1]. These siblings constitute the first reported cases of GABA‐transaminase deficiency secondary to a homozygous nonsense variant of the *ABAT* gene, and hints to a possible correlation between presumptive complete loss of function and a more clinically severe phenotype. Additionally, the finding of a truncating, likely pathogenic variant *ABAT*:c.1297del (p.Arg432_Val433insTer) reported downstream of our reported variant provides further evidence of pathogenicity of our variant [6].

We suspect the nonsense variant described here may be a founder mutation in the Canadian Indigenous community from which this family originates. Despite no reported consanguinity, our patients were homozygous for a unique, previously unreported variant. In addition, the two previously affected siblings in this family both had chromosomal SNP microarrays that showed a multitude of small regions of homozygosity, consistent with both parents coming from a founder population (data not shown). Figure 4b shows a small region (~4.5 Mb) of homozygosity surrounding the *ABAT* gene in both of these sibs. In our clinical experience, regions of this size are generally associated with distant parental relationships and suggest carrier status may have an appreciable frequency in this population. There is currently no population genomic database that captures genomic data from this Canadian Indigenous community. Further evidence is needed to prove this but given the early lethality, this diagnosis should be considered early in the work‐up of any severely neurologically impaired neonate with this ethnic background.

Many cases of GABA‐transaminase deficiency have reported laboratory findings of elevated GABA in the plasma and CSF [7, 8, 9]. While previous cases have reported higher concentrations of GABA in urine, the exact quantification is not known, and no established reference ranges are available [3]. Despite this, the elevation of urinary GABA was so significant in our index case that we propose future biochemical testing include GABA measurements in the urine. This may be an easier alternative to subjecting the patients to a lumbar puncture for GABA levels in the CSF, although the sensitivity of quantification of GABA from different tissues is yet to be determined. Additionally, a recent study performed metabolomic analyses of over 1000 clinical plasma samples in four patients, and reported increased levels of 2‐pyrrolidinone in the plasma, as well as increased levels of succinimide in the plasma, urine and CSF in three out of four patients (*R* = 0.72), while GABA was not detected in the plasma [10]. The raised plasma 2‐pyrrolidinone and succinimide levels are due to downstream effects of GABA accumulation, and suggests that these analytes may be helpful depending on when biochemical workup was initiated during the course of this illness. However, the authors also noted that the levels of 2‐pyrrolidinone may be affected by antiseizure medications like vigabatrin or topiramate [10].

Flumazenil is a competitive inhibitor of the benzodiazepine binding site of the GABA^A^ receptor [11]. Continuous flumazenil infusion trials have previously been implemented on two patients with GABA‐transaminase deficiency. In one patient, his phenotype was milder with seizure onset at 6 months of age [1, 7]. Flumazenil was given continuously for 20 months, which showed an improvement in background organization on EEG, as well as a reduction in choreoathetosis and myoclonic movements [7]. The second patient had severe developmental delay and was trialed on flumazenil infusion for 2 months without success [5]. Unfortunately, our index patient had a severe clinical presentation with electrocerebral silence on EEG prior to the infusion of flumazenil. She had slight improvement of her EEG within 24 h of infusion and subtle clinical improvement, although we may have seen further improvement over time. Further flumazenil trials in cases of GABA‐transaminase deficiency are warranted with the caveat that it may have limited utility in more severe cases or those later on in the course of disease progression. While prognosis has been guarded in most cases, the first patient with GABA‐transaminase deficiency who survived into adulthood has been reported [12]. At the age of 21, however, he continues to have seizures that have been controlled with lamotrigine and sodium valproate and is entirely dependent in his activities of daily living. It is not known if this patient was ever trialed on flumazenil.

Furthermore, MRI brain findings of cerebellar vermis agenesis and hemispheric hypoplasia observed in the index patient and her affected siblings were previously reported in one patient with GABA‐transaminase deficiency. Our report indicates the likely possibility of a unique neuroimaging phenotype, with predominant cerebellar abnormalities, relating to this disorder. Despite being started on cPAP from birth, the proband had persistent respiratory acidosis, which required subsequent intubation. This may have contributed to the lactate peak on MRS.

## 4 Conclusion

We report three additional cases of GABA‐transaminase deficiency occurring within the same family. They each presented with a severe clinical phenotype and characteristic neuroimaging findings raising the possibility in the association between homozygous nonsense *ABAT* genotype and a more severe form of the disease. A therapeutic trial with flumazenil infusion was attempted but unsuccessful in the index patient. Further exploration into the utility of GABA measurements in urine should be explored, with proper reference ranges to be established.

## Author Contributions

Data collection: Deima Alammary, Tisiana Low, Marie‐Claude Dery, Mahmoud Almutadares, Samantha Marin, Mubeen F. Rafay, and Katya Rozovsky. Literature review: Tisiana Low and Patrick Frosk. Draft manuscript preparation: Deima Alammary, Tisiana Low, Samantha Marin, Mubeen F. Rafay, Katya Rozovsky, and Patrick Frosk. Interpretation: Tisiana Low, Samantha Marin, Mubeen F. Rafay, Katya Rozovsky, and Patrick Frosk. Supervision: Patrick Frosk. All authors reviewed the results and approved the final version of the manuscript.

## Funding

The authors have nothing to report.

## Ethics Statement

Patient consent: signed consent was obtained from the parent of the index patient.

## Conflicts of Interest

The authors declare no conflicts of interest.

## Data Availability

The data that support the findings of this study are available from the corresponding author upon reasonable request.
